# An Analysis Pipeline for Genome-wide Association Studies

**DOI:** 10.4137/cin.s966

**Published:** 2008-09-24

**Authors:** Stefan Stefanov, James Lautenberger, Bert Gold

**Affiliations:** 1 Human Genetics Section, Laboratory of Experimental Immunology, National Cancer Institute at Frederick, Frederick, MD 21702; 2 Laboratory of Genomic Diversity, National Cancer Institute at Frederick, Frederick, MD 21702

**Keywords:** single nucleotide polymorphism, SNP, genetic association, GWAS, genetic epidemiology

## Abstract

We developed an efficient pipeline to analyze genome-wide association study single nucleotide polymorphism scan results. Purl scripts were used to convert genotypes called using the BRLMM algorithm into a modified PB format. We computed summary statistics characteristic of our case and control populations including allele counts, missing values, heterozygosity, measures of compliance with Hardy-Weinberg equilibrium, and several population difference statistics. In addition, we computed association tests, including exact tests of association for genotypes, alleles, the Cochran-Armitage linear trend test, and dominant, recessive, and overdominant models at every single nucleotide polymorphism (SNP). In addition, pairwise linkage disequilbrium statistics were elaborated, using the command line version of HaploView, which was possible by writing a reformatting script. Additional Perl scripts permit loading the results into a MySQL database conjoined with a Generic Genome Browser (gbrowse) for comprehensive visualization. This browser incorporates a download feature that provides actual case and control genotypes to users in associated genomic regions. Thus, re-analysis “on the fly” is possible for casual browser users from anywhere on the Internet.

## Introduction

Genome-wide polymorphism and copy number variation arrays are an accepted and standardized approach to disease association mapping, which is gaining in popularity in the genetic research community. Nearly two hundred genome-wide association studies (GWAS) have now been cataloged by the National Human Genome Research Institute of the National Institutes of Health (NIH; see: http://www.genome.gov/GWAstudies/). In addition, newer platforms provide detailed typing for both common and rare copy number variations (CNVs) in the human genome.

Before mid-2007, a 500K Affymetrix array was the most common platform used to study the genetic epidemiology of cancer and to help understand the population genetics of common diseases. Since that time, Illumina has released a 550 K, 650 K and a One Million (1 M) single nucleotide polymorphism (SNP) platform; and Affymetrix has released a 6.0 version that contains over 900,000 SNPs (with over 700,000 scorable) and approximately 950,000 CNV probes. Each platform provides copious genetic data on every typed research subject.

The mass application of the 500 K array around the world has produced an enormous amount of data susceptible to independent and creative analysis in many locations. Both population history studies and association analysis can be carried forward with these data. The ease and availability of analysis can contribute immensely into gaining new insights from the data provided from dense SNP and CNV panels.

A methodologic pipeline for analysis of the microarray genotype calls, performed directly on a Unix platform, using an average PC-Linux or Mac computer, avoids cumbersome and time-consuming statistical software programming and permits rapid conclusions to be drawn about population structure and associations. Here, we provide methodology and detailed instructions for implementing the analysis of GWAS results.

## Results

### Construction of a single file in modified PB format

The main feature of our analytical pipeline is the concentration of all available information into a single text file. We call it a modified PB file. This single file comprises structured SNP calls, confidence scores, and references for all mapped SNPs for hundreds or thousands of genotyping microarrays.

Our modified PB format is based on the Prettybase format developed by the SeattleSNPs project (http://pga.gs.washington.edu) to easily represent SNP information for groups of subjects over a given span of reference sequences. Our modified PB format has an inherent feature of easy adaptability to the descriptive applications and tools written by Ross Lazarus and publicly available at http://innateimmunity.net.

**Figure 1:**
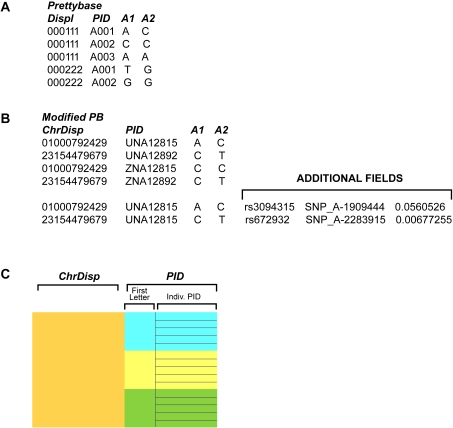
Input File Formats. **A**) Schematic structure of a traditional Prettybase file with the four columns delineated. The leftmost column is the displacement relative to a reference value; the middle column is a patient identifier (PID); and the third and fourth columns are the allele 1 and allele 2 base calls (A1 A2). **B**) A modified format designated as a Modified PB file. The leftmost displacement file consists of an 11 digit displacement, the first two digits of which are the chromosome number (X is 23, Y is 24), and the rightmost 9 digits of which are the displacement relative to the beginning of the chromosome. The PID consists of alphanumeric identifiers preceded by a group identifier (for example, U for Utah or Z for Zuni Native American) with the third and fourth columns as allele 1 and allele 2 base calls (A1 A2). Additional fields appended to the right hand portion may include dbSNP identifiers, Affymetrix probe identifiers, or confidence values, as shown in the right hand portion of panel B. **C**) Cartoon clarifying the relationship of the 11 digit chromosome-displacement identifier (ChrDisp) with the population subgroup PID identifier, ordered through a UNIX sort.

The following Prettybase features were adapted to our modified PB format: (Fig. 1, Panel B):

1. The file consists of rows with four fields separated by a white space. These fields are: SNP displacement, Patient Identifier (PID), genotype call for the first allele, and genotype call for the second allele.
2. A missing genotype, if present, is denoted N N in the third and fourth columns.
3. The first letter of a PID indicates the population to which this subject belongs.

We added some other characteristics to the Prettybase format, thus rendering it into the modified PB format. The first of these characteristics is the 11-digit chromosome-displacement field (ChrDisp) specifying the unique position of the SNP in the human genome. The first two digits contain the chromosome number padded with leading zeros, if necessary, while the last nine digits contain the base pair position (again padded with leading zeros). [See Fig. 1] Also, a modified PB file can have as many fields as necessary: While the four core fields do not alter relative position, additional fields containing meta-data can be appended on the right. Adding these fields may include new information but does not complicate the reading or parsing of the file for reformatting or computation. In modified PB format (Fig. 1, Panel B), fields for dbSNP RefSNP ID, Affymetrix SNP ID, and confidence score of genotyping have been added.

The modified PB file can be sorted with a simple “sort” command (UNIX, Perl or Excel type), which will group the record lines into blocks of the same SNP, sorted according their position in the genome. Each block, in turn, is sorted according to the PID (Fig. 1, Panel C).

This modified PB file can have different numbers of fields and lines; a variety of chromosome-displacements or population specifications. Thus, we define a “regular” modified PB as a file that contains an equal number of persons per SNP block and equal number of fields per record. In general, we always refer to that type of modified PB file, since the number of the records equals the product of the number of SNPs multiplied by the number of genotyped research subjects.

### Affymetrix 500K and Illumina GoldenGate analysis using modified PB file format and minimum software

**Figure 2:**
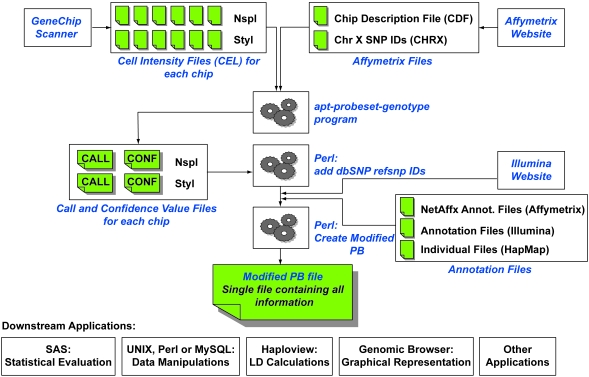
Analysis Pipeline Workflow. The pipeline was initially written for the analysis of Affymetrix genotyping microarrays, but has since been adapted to Illumina Bead Arrays. The upper portion of the figure provides a workflow for dual style (500 K) microarrays, but has also been used for single-chip Affy 6.0 microarrays. Illumina BeadArray 317 K, 500 K, 550 K, 650 K and 1 M data feeds into the workflow on the right-hand side, after flat table export of a BeadStudio V3.x “Full Data Table Output” file. The arrays provide a basis for assembly of the modified PB file, which is the raw input for the analytic pipeline.

The modified PB-file pipeline is used in our laboratory and is the backbone for a recent study of genetic variation in Ashkenazi Jews (Olshen et al. 2008) and one recently published GWAS (Gold et al. 2008) (Figure 2).

Two sources of input data were provided to us from Affymetrix 500 K scans:

1. Data: CEL files from the Affymetrix GeneChip reader from a genotyping facility: The CEL files are binary files containing the fluorescence intensities for each probe on the microarray. Those files together with SNP-specific annotation files obtained from the Affymetrix website were processed in two steps into a modified PB file within a few hours.
  Step 1: Running BRLMM genotyping algorithm
  A command line program called *apt-probeset-genotype* was used. It is part of the open-source Affymetrix Power Tools (APT) software package (Affymetrix, 2008a). This is an application for making genotype calls from mapping arrays. The application supports the BRLMM genotype calling algorithm (a modification of Rabbee and Speed, 2006). The user can select different outputs including SNP calls and confidence scores. All SNP values are tagged with SNP Affymetrix ID.
  Step 2: Converting retrieved data into the modified PB format
  SNP Affymetrix IDs in the output data were mapped with their respective dbSNP RefSNP IDs (through a Perl script or MySQL) or were discarded. It was important to use dbSNP identifiers instead of Affymetrix IDs for further processing with annotations. While each SNP is undoubtedly identified by its dbSNP ID, it can have more than one Affymetrix ID depending upon the probeset used to detect it. Thus, different Affymetrix IDs on different chip releases may detect the same dbSNP ID. For Affymetrix genotyping microarrays, a Perl script was used to convert all data into a single modified PB file with seven columns.
  Optionally, a LINKAGE-format individuals’ file could be used to select for PIDs.
2. Annotations: A NetAffx annotation information file that provides consolidated data from multiple public sources, including probe sequences, gene annotations, and extensive annotation for each SNP (Affymetrix, 2008b) is required for further processing.

For the processing of Illumna GoldenGate or Infinium data, A Perl script was constructed that permitted flat BeadStudio V3.x “Full Data Table Output” files to be converted to modified PB format through accurate parsing and reformatting.

### Statistical analysis using modified PB files and SAS

SAS software (SAS Institute Inc., Cary, NC) version 9.1.3, running on a SUSE Linux platform, converted the PB files into SAS data sets for further analysis. Commands and instructions were written according to the *Language Reference* (SAS Institute, 2004a) and *Procedures Guide* (SAS Institute, 2004b) and are available at the authors’ FTP site (ftp://ftp.ncifcrf.gov/pub/users/goldb).

The genotypes were recoded so that the most common allele in a reference population (reference allele) was assigned a value of 0 and the other allele (variant allele) was assigned a value of 1. For each marker in each population summary statistics were computed that included the number of observed alleles (1 or 2), the allele and genotype counts and frequencies, the Hardy-Weinberg (H-W) disequilibrium coefficient (*D*), inbreeding coefficient (*F*_IS_), and the test statistic and *P-value* for the chi-square test for Hardy-Weinberg equilibrium. The genotype counts were used to compute exact *P-values* by the method of Wiggington et al. (2005). The direction of H-W deviation was also assessed.

Several genetic distance statistics were computed for the populations including the Fixation Index of the Subpopulation within the Total (*F*_ST_) (Weir, 1996) and Nei’s standard genetic distance measure (*D*_S_) (Nei, 1972, 1978). In addition, several information theory-based statistics were computed, including entropy for admixed populations (Smith et al. 2003), Kullback-Leibler divergence (Cover and Thomas, 1991) and the informativeness (I_n_) for assignment statistic of Rosenberg et al. (2003).

Three chi-square tests comparing marker allele and/or genotype frequencies were implemented with SAS software. They consist of the allele test (Pearson chi-square test on table of allele counts by disease status), the genotype test (Pearson chi-square test on table of genotype counts by disease status), and the trend test (Armitage test on table of genotype counts by disease status). Equivalent exact tests were implemented through SAS PROC FREQ. In addition, Fisher’s exact test was used to test recessive and dominant models. Odds ratios were computed for the allele test by PROC ALLELE and for the trend test by PROC LOGISTIC. The genetic distance and association statistics are summarized in a table with one line per SNP. (Supplemental Table 1).

## Discussion

Genome-wide association studies require team-work between disparate centers, laboratories, and technologies. Samples located in one center may be received in another, typed in a third and analyzed in a fourth location, often across continents. Today, in spite of rapid Internet connections, the transfer of hundreds of gigabytes of row genotype data can be a serious time and security challenge. While the newest hardware configurations of PC and Mac computers could be found in nearly every research institution, this is not the case with expensive proprietary software, SNP genotyping arrays or medical samples. Therefore, we proposed a method for standard assembly and quick extraction of genotype information from row data. This method provides easy transportability, a standardized, easily understandable and robust format that would be advantageous for any SNP-typing or mapping association study.

Our analytic platform allows collection of a huge amount of data in a short amount of time. Thus, the modified PB file can be promptly forwarded for further statistical analysis.

As part of our effort, Perl scripts were written which converted and supplied the modified PB for analysis using SAS, Haploview, PHASE and the R platform. This permitted the generation of three different types of statistics from the data assembled in the PB:

1. Population differentiation statistics: Fixation index (*F*_ST_), Entropy index, Nei’s Gene substitution Distance Measure (*D*_S_), Kullback-Leibler Average Distance measure, Informativeness for assignment (I_n_), etc.;
2. H-W Compliance and Call consistency measure statistics used to measure the quality of genotyping;
3. Association statistics: allele and genotype frequencies, trends, odds ratios, confidence intervals, haplotypes for associations.

In addition, we have implemented Perl scripts that permit calculation of statistics that report the consistency of our data with HapMap data and consistency of segregation in families. These statistics provide a basis for quality assurance of the typing data.

We have compared our GWAS analysis pipeline with the PLINK package authored by Purcell and his collaborators (Purcell et al. 2007). PLINK provides an outstanding collection of software routines aimed at GWAS analysis; however, its extensive association testing capabilities do not provide a uniform output format. In fact, each PLINK command yields a novel output, some organized by SNP, some by SNP group or sliding window, some by CNV, or by PID. Instead, we chose a method that provides a uniform output format with 112 columns or fields that could be uploaded into a database or browser for further analysis. In addition, several statistics are available through our pipeline that are not available through PLINK: Among these are some association statistics, such as the co-dominant and overdominant models, an elaboration of Hardy-Weinberg compliance statistics (Weir’s *D*-coefficient for the direction and magnitude of H-W deviations [Weir, 1996]), and several statistics that describe differences in populations through allele frequency calculations. The latter include *F*_ST_ between the ostensible case (S in first PID column) and control (L in first PID column) populations(Weir, 1996 equation 5.2, p. 173), entropy with a 10%, 50% or 90% control ancestry calculation (Smith et al. 2004, equation 1), the Kullback-Leibler divergence in both directions averaged (Rosenberg et al. 2003 at page 1403 a factor of 1/2 used; see note c) and a Kullback-Leibler divergence—a “resistor” average (Johnson et al. 2001, equation 11); In, informativeness for assignment (Rosenberg et al. 2003, equation 4) and Nei’s *D*_S_ or standard genetic distance (Nei, 1972; Takezaki and Nei, 1996; equation 1).

The central element of our analysis is the modified PB text file. This single file summarizes all data from the genotype calls and confidence scores of many chips and individuals (grouped into populations). The file can be zipped and transported, and may be used as input for many types of analysis. Furthermore, with the wide availability of hard disks of 1.5 terabyte capacity, data may be analyzed all at once on PC-Linux and Macintosh computers using only UNIX, Perl, MySQL, R or other General Public License (GPL) software. This will permit users to avoid high-performance hardware and expensive proprietary software. The file can also be parsed and dispersed into 24 chromosome files (25 if mitochondrial variants are included) for individual chromosomal analysis. In our experience, although the Windows XP operating system is user-friendly compared to UNIX, the restrictions it puts on the size of input data for its basic applications (e.g. ~65,000 lines for Excel) render it impractical for this volume of data analysis.

On a Linux PC with 2 gigabytes of Random Access Memory (RAM), the process of building a modified PB file from Affymetrix 500 K CEL files for few hundred individuals may take 1–2 hours. This time estimate accounts for a minimum of other processes running on the PC. Although this is an imprecise performance measure, this processing time frame estimate should provide potential users with a “rule of thumb guide” for processing time requirements of the pipeline we have described.

## Supplementary Material

Table S1
